# Azithromycin attenuates cigarette smoke extract-induced oxidative stress injury in human alveolar epithelial cells

**DOI:** 10.3892/mmr.2015.3226

**Published:** 2015-01-20

**Authors:** MIAOMIAO CHEN, TUO YANG, XIANGIYU MENG, TIEYING SUN

**Affiliations:** 1 Department of Respiratory and Critical Care Medicine, Tianjin Chest Hospital, Tianjin 300000, P.R. China; 2 Department of Respiratory and Critical Care Medicine, Fifth School of Clinical Medicine, Peking University, Beijing Hospital Ministry of Health, Beijing 100730, P.R. China; 3 Department of Respiratory and Critical Care Medicine, Beijing Hospital Ministry of Health, Beijing 100730, P.R. China

**Keywords:** cigarette smoke extract, reactive oxygen species, azithromycin

## Abstract

Cigarette smoking has been verified to be one of the most important etiological factors causing the development of bronchogenic carcinoma and chronic obstructive pulmonary disease. Azithromycin (AZM) has been demonstrated to have antioxidant capacity. In the present study, whether AZM is able to attenuate cigarette smoke extract (CSE)-induced A549 cell oxidative stress injury was investigated. Cells were incubated with CSE in the presence or absence of AZM. Cell viability was measured using a 3-(4,5-dimethylthiazol-2-yl)-2,5-diphenyltetrazolium bromide assay. The expression of vascular endothelial growth factor (VEGF) was analyzed using western blotting and ELISA. The expression of epithelial cell structural proteins, zona occludens (ZO)-1 and occludin was determined using western blotting and immunofluorescence staining. Reactive oxygen species (ROS) production was examined by flow cytometry and fluorescence staining. The results demonstrated that the exposure of A549 cells to CSE decreased cell viability in a dose- and time-dependent manner. AZM significantly attenuated the CSE-induced decreases in the expression of VEGF and epithelial cell structural proteins, including ZO-1 and occludin. CSE also stimulated ROS production in the A549 cell, while AZM significantly reversed the effects of CSE. In addition, the inhibition of ROS by N-acetyl-L-cysteine had similar effects as AZM on the expression of VEGF and epithelial cell structural proteins and also enhanced cell proliferation. In conclusion, AZM attenuated CSE-induced oxidative stress injury in A549 cells and may be a promising therapeutic agent for smoking-associated pulmonary diseases.

## Introduction

Cigarette smoking is not only one of the most severe public health issues but also one of the most supported etiological factors contributing to the development of bronchogenic carcinoma and chronic obstructive pulmonary disease (COPD) (1). Cigarette smoke (CS) is a complex combination of chemicals, including high levels of oxidants. Alveolar epithelial cells (AECs) appear to be a major target for oxidant injury of the various cell types of the lung (2–4). Injury of the alveolar epithelium by CS is hypothesized to be an important process in the pathogenesis of smoking-associated pulmonary diseases. Therefore, protection of AECs from injury by CS appears to be crucial for the management of numerous lung diseases associated with cigarette smoking.

The breach in epithelial integrity may constitute one of the earliest elements of lung injury in response to CS exposure (5–7). Breaches of the epithelial barrier may induce wound-repair responses and a persistent increase in epithelial permeability. This may lead to the subepithelial tissue being directly exposed to CS. Oxidative stress and active cytoskeletal rearrangement are critical for the development of epithelial barrier disruption induced by CS (8,9). Decreases in the levels of zona occludens (ZO)-1 and occludin are associated with epithelial barrier dysfunction and increased epithelial permeability (10,11). Accumulation of occludin and ZO-1 at tight junctions (TJs) is associated with protection of the epithelial barrier (12). Occludin is anchored to the cytoskeleton by ZO-1 (13). In the process of abnormal epithelial repair, the expression of ZO-1 and occludin decreases (14), which causes epithelial cells to lose their normal structure. ZO-1 and occludin are two well-characterized proteins in TJs, which are associated with epithelial barrier structure and integrity (15). CS causes the delocalization of ZO-1 and occludin from the cell-cell boundaries and a subsequent loss of epithelial integrity (16).

Smoking not only affects epithelial cell structure, but also various functions of epithelial cells, including synthesis and secretion. Vascular endothelial growth factor (VEGF) is a potent angiogenic protein that has been implicated in a number of structural and functional alterations of vascular endothelial cells. The survival of endothelial cells is largely achieved through the action of VEGF, which is abundantly expressed in the lung (17). VEGF has autocrine effects on the growth and proliferation of pulmonary epithelial cells *in vitro* (18). VEGF is predominantly secreted by AECs and cigarette smoke extract (CSE) reduces VEGF production in epithelial cells (19). Decreased levels of VEGF are known to occur in smokers, in the lungs of patients with COPD and in rat lungs in response to CS exposure (20,21). As a result, inhibiting these decreases in VEGF induced by CSE may prevent emphysema development.

Macrolides are a group of antibiotics that are characterized by a macrocyclic lactone ring with various amino sugars attached. In addition to their antimicrobial activity, a number of these antibiotics also have immunomodulatory properties, as demonstrated in multiple *in vitro* and *in vivo* studies (22–24). The immunomodulatory effects are associated with the lactone ring, which is only observed in the 14 (erythromycin, clarithromycin and roxithromycin) and the 15 (azithromycin, AZM) membered macrolides (25). AZM is different from other macrolide antibacterial drugs in that it possesses unusual pharmacokinetic properties. It accumulates at a high rate in cells and tissues and has a plasma half-life of >40 h (26). In the airway epithelial cells and neutrophils of cystic fibrosis patients, AZM has been demonstrated to have antioxidant capacity (27–29). However, there have been no studies, to the best of our knowledge, investigating the possible role of AZM in the protection of human AECs from oxidative injury induced by CSE. The present study examined the effect of AZM on the regulation of CSE-induced injury in the human alveolar epithelial cell line A549.

## Materials and methods

### Cell culture and drug treatment

Cell cultures of the A549 human type II alveolar epithelial cell line (Cell Research Center, Institute of Basic Medical Sciences, Chinese Academy of Medical Sciences, Beijing, China) were grown in Dulbecco’s modified Eagle’s medium (DMEM)/F-12 culture medium (HyClone, Logan, UT, USA) containing 10% heat-inactivated fetal calf serum (HyClone), 100 kU/l penicillin and 100 mg/l streptomycin (Invitrogen Life Technologies, Carlsbad, CA, USA). The cells were maintained at 37°C in a humidified atmosphere at 5% CO_2_. The cell cultures were maintained until they were ~70–80% confluent and subsequently incubated in serum-free DMEM (SF-DMEM) for 16 h. AZM (Zithromax; Pfizer Pharmaceuticals, Dublin, Ireland) and N-acetyl-L-cysteine (NAC) were dissolved in sterilized phosphate-buffered saline (PBS; Zhongshan Biotechnology, Beijing, China). Prior to incubation with or without CSE, AZM and NAC were added to cells for 2 h.

### Preparation of CSE

Fresh CSE was prepared for each experiment. Briefly, one commercial filtered cigarette (Derby; China Tobacco Anhui Industrial Co., Ltd, Hefei, China) was passed through 10 ml of preheated DMEM using a peristaltic pump, with the pH adjusted to 7.4, and subsequently filtered through a 0.22 mm filter. The absorbance of 320 nm measured using a Hitachi U-3900H (Hitachi High-Technologies, Tokyo, Japan)revealed few differences between different preparations of CSE. The solution was considered 100% CSE and was diluted for each experiment.

### Assay of A549 cell viability

The viability of the A549 cells was determined using a colorimetric, 3-(4,5-dimethylthiazol-2-yl)-2,5-diphenyltetrazolium bromide (MTT) assay (Sigma-Aldrich, St. Louis, MO, USA). Briefly, the cells were cultured in 96-well tissue culture plates, grown to 70–80% confluence and subsequently incubated for over 16 h in SF-DMEM F-12 medium. After 24 h CSE treatment, the cells were incubated with 0.5 mg/ml MTT in fresh medium for a further 4 h. The blue formazan was dissolved by adding dimethyl sulfoxide (Sigma-Aldrich)and was spectrophotometrically measured at a wavelength of 570 nm using a Thermo Scientific Multiskan FC (Thermo Fisher Scientific, Waltham, MA, USA).

### Protein preparation and western blot analysis

Following incubation, the cells were washed with ice-cold PBS twice. Proteins were extracted from the A549 cells using radioimmunoprecipitation assay buffer [50 mM Tris/HCl, pH 7.4, 150 mM NaCl, 1% (v/v) NP-40, 0.1% (w/v) SDS; Solarbio Science and Technology Co., Ltd., Beijing, China] containing a protease inhibitor cocktail (AEBSF, bestatin, E-64, leupeptin, pepstatin A and 1,10-phenanthroline; catalogue number, P9599; 100:1, v:v; Sigma-Aldrich). The cell lysates were subjected to centrifugation at 12,000 × g at 4°C for 15 min and the supernatant was collected as total protein. Protein quantitation was performed using the bicinchoninic acid method according to the manufacturer’s instructions (Pierce Biotechnology, Inc., Rockford, IL, USA). The samples were separated on 10% SDS-polyacrylamide gels and transferred onto a polyvinylidene difluoride membrane (Millipore, Billerica, MA, USA), which was soaked in 5% milk in Tris-buffered saline with Tween 20 (TBST; pH 7.6; Sigma-Aldrich) for 2 h at room temperature. The membrane was incubated overnight with a rabbit VEGF monoclonal antibody (1909-1; Epitomics, Burlingame, CA, USA) at a dilution of 1:250, a rabbit ZO-1 polyclonal antibody (ab59720; Abcam, Cambridge, UK) at a dilution of 1:100 or a rabbit polyclonal antibody occludin (71-1500; Invitrogen Life Technologies) at a dilution of 1:250 on a rotating platform at 4°C. Following washing with TBST (pH 7.6), the membranes were incubated in horseradish peroxidase-conjugated goat anti-rabbit and mouse IgG, at a 1:5,000 dilution (M21003; Abmart, Shanghai, China) for 2 h on a rotating platform at room temperature (RT). The antibody complexes were detected by chemiluminescence (Millipore) according to the manufacturer’s instructions. Bands were quantified using a PhosphorImager and ImageQuant software version 1.46 (Amersham Biosciences, Amersham, UK) and normalized to GAPDH.

### Immunofluorescence

Cells cultured on six-well chamber slides were washed with PBS three times for 5 min per wash and fixed in 4% paraformaldehyde for 30 min at RT. Following a further three washes with PBS for 5 min per wash, the slides were incubated with 3% bovine serum albumin (BSA; Sigma-Aldrich) in PBS for 1 h at RT. Subsequently, cells were incubated with primary antibodies against human ZO-1 and occludin, and diluted at 1:100 in PBS with 1% BSA. Following incubation with the primary antibodies overnight at 4°C, the cells were washed with PBS and incubated with Alexa Fluor 488-conjugated anti-rabbit IgG (ZF-0511; Zhongshan Biotechnology Co., Ltd., Beijing, China; diluted at 1:50 in PBS with 1% BSA) for 1 h at RT. Following three washes in PBS, the slides were stained with 10 μg/ml Hoechst 33258 for 10 min at RT. The slides were washed again and mounted. Immunofluorescence images were captured by fluorescence microscopy (Eclipse 80i; Nikon Corp., Tokyo, Japan).

### Fluorescence staining

Cells cultured on six-well chamber slides were washed with PBS three times for 5 min per wash and subsequently incubated with ROS Fluorescent Probe-dihydroethidium (DHE; Vigorous Biotechnology Beijing Co., Ltd., Beijing, China) in SF-DMEM F-12 medium for 30 min at 37°C in darkness and fixed in 4% paraformaldehyde for 30 min at RT. The slides were washed again and mounted. Immunofluorescence images were captured by fluorescence microscopy (Eclipse 80i; Nikon Corp.).

### Quantification of intracellular ROS

Intracellular ROS was measured using ROS Fluorescent Probe-DHE. Following a 24 h treatment of CSE or 200 μM H_2_O_2_, the cells were washed with PBS and subsequently incubated with 5 μM DHE in PBS for 30 min at 37°C in darkness. The cells were harvested and washed with PBS. Following centrifugation at 800 × g for 6 min, the cells were suspended in PBS. Relative fluorescence intensities in the A549 cells were analyzed with flow cytometry (FACS Calibur; BD Biosciences, Franklin Lakes, NJ, USA).

### ELISA

Following pretreatment with AZM and incubation with CSE for 24 h or 10% CSE for 6, 12, 24 or 48 h, the cell culture medium was centrifuged at 1006.2 × g for 20 min and subsequently the supernatant was collected. Quantification of VEGF levels was performed using human VEGF ELISA kits (Bio-rexd, Beijing, China) according to the manufacturer’s instructions.

### Statistical analysis

All data are expressed as the mean ± standard error of the mean. The statistical significance of the differences was evaluated by analysis of variance and subsequently by Tukey’s multiple-comparison procedure. P<0.05 was considered to indicate a statistically significant difference.

## Results

### CSE affects A549 cell viability in a dose- and time-dependent manner

**Figure 1:**
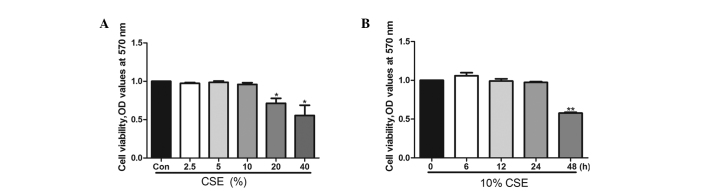
A549 cell viability is affected by CSE in a dose- and time-dependent manner. (A) A549 cells were exposed to 2.5, 5, 10, 20 and 40% CSE for 24 h. (B) A549 cells were exposed to 10% CSE for 6, 12, 24 and 48 h. Exposing A549 cells to CSE affected the cell viability in a dose- and time-dependent manner. The data are presented as the mean ± standard error of the mean of three independent experiments. ^*^P<0.05 and ^**^P<0.01 versus controls. CSE, cigarette smoke extract; OD, optical density.

A549 cells were exposed to 2.5, 5, 10, 20 and 40% CSE for 24 h or to 10% CSE for 6, 12, 24 and 48 h. A549 cell viability was decreased when the cells were treated with 20% CSE for 24 h (Fig. 1A) or with 10% CSE for 48 h (Fig. 1B). Following 24 h of incubation, CSE at concentrations of 10% did not affect cell viability. In the subsequent experiments, CSE was used at a concentration of 10% for 24 h.

### CSE attenuates the expression of VEGF in a dose- and time-dependent manner

**Figure 2:**
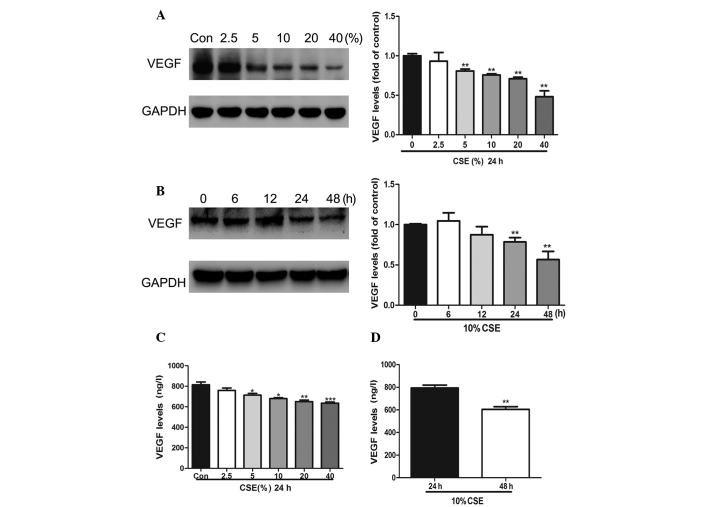
CSE attenuates VEGF expression in a dose- and time-dependent manner. (A and C) A549 cells were treated with 2.5, 5, 10, 20 and 40% CSE for 24 h or (B) 10% CSE for 6, 12, 24 and 48 h. (A) VEGF protein expression was decreased by CSE in the A549 cells in a dose-dependent manner. (B) VEGF protein expression in the A549 cells decreased significantly by 24 or 48 h. Furthermore, the secretion of VEGF in the culture medium was measured. (C and D) VEGF secretion was decreased by CSE in a dose- and time-dependent manner. The data are presented as the mean ± standard error of the mean of three independent experiments. ^*^P<0.05 and ^**^P<0.01 versus controls. CSE, cigarette smoke extract; VEGF, vascular endothelial growth factor.

In order to determine the effects of CSE on VEGF protein expression, A549 cells were treated with 2.5, 5, 10, 20 and 40% CSE for 24 h (Fig. 2A and C) or with 10% CSE for 6, 12, 24 and 48 h (Fig. 2B) or 10% CSE for 24 and 48 h (Fig. 2D). Western blot analysis and ELISA demonstrated that the CSE treatment significantly reduced the levels of VEGF expression in a dose- and time-dependent manner.

### CSE attenuates the expression of ZO-1 and occludin in a dose- and time-dependent manner

**Figure 3:**
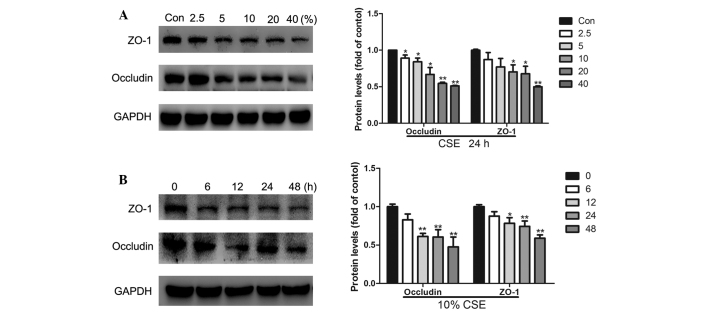
CSE attenuates expression of ZO-1 and occludin in a dose- and time-dependent manner. (A) A549 cells were treated with 2.5, 5, 10, 20 and 40% CSE for 24 h. ZO-1 and occludin protein expression in the A549 cells decreased significantly after 12, 24 or 48 h. (B) A549 cells were treated with 10% CSE for 6, 12, 24 and 48 h. ZO-1 and occludin protein expression decreased following exposure to CSE in the A549 cells in a dose-dependent manner. The data are presented as the mean ± standard error of the mean of three independent experiments. ^**^P<0.01 versus controls. CSE, cigarette smoke extract; ZO-1, zona occludens-1.

The expression of ZO-1 and occludin was investigated using western blotting. A549 cells were treated with 2.5, 5, 10, 20 and 40% CSE for 24 h or with 10% CSE for 6, 12, 24 and 48 h. Western blot analysis revealed that CSE treatment significantly reduced the levels of ZO-1 and occludin expression in a dose- and time-dependent manner (Fig. 3). At doses of 2.5, 5, 10, 20 and 40%, the decreases in occludin protein expression were 11, 16, 33, 45 and 49% and at doses of 10, 20 and 40%, the decreases in ZO-1 protein expression were 30, 32 and 50%, respectively (Fig. 3A). When the A549 cells were treated with 10% CSE for 12, 24 and 48 h, occludin protein expression decreased by 39, 40 and 52% and ZO-1 protein expression decreased by 22, 26 and 42% (Fig. 3B).

### AZM suppresses CSE-induced ROS increase in A549 cells

**Figure 4:**
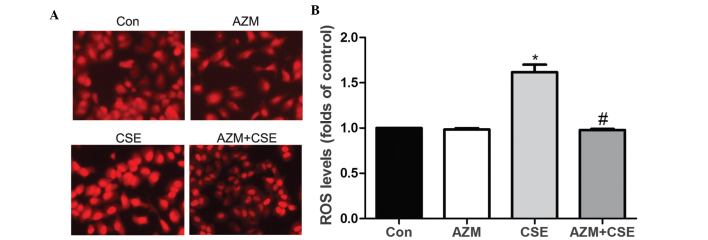
AZM suppresses CSE-induced ROS increases in A549 cells. (A) Cells stained with ROS Fluorescent Probe-DHE to indicate levels of ROS (magnification, ×200). (B) Quantification of ROS increase in the control and treated cells. AZM suppressed CSE-induced ROS increases in A549 cells. n=3 independent experiments. ^*^P<0.05 versus controls; ^#^P<0.05 versus CSE. CSE, cigarette smoke extract; AZM, azithromycin; ROS, reactive oxygen species; Con, control.

To determine whether AZM affected CSE-induced ROS increases in A549 cells, the cells were divided into four groups, including a control group, an AZM group, a CSE group and a CSE-supplemented AZM group (CSE+AZM). The A549 cells were pretreated with AZM (10 μg/ml) for 2 h and subsequently treated with 10% CSE for 24 h. Fluorescence staining (Fig. 4A) and flow cytometry (Fig. 4B) illustrated that A549 cells incubated with CSE had significantly increased ROS generation. ROS generation induced by CSE was decreased by AZM in the A549 cells.

### AZM reverses the alterations in VEGF, ZO-1 and occludin induced by CSE

**Figure 5:**
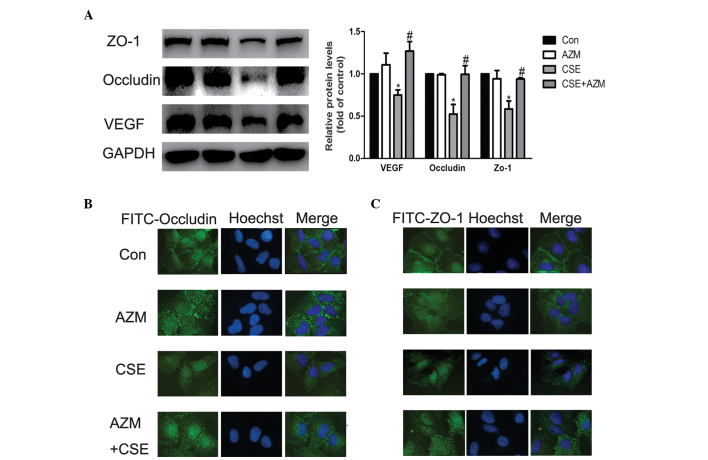
AZM suppresses CSE-induced injury in A549 cells. (A–C) Exposure of A549 cells to CSE significantly decreased occludin and ZO-1, while AZM inhibited these changes induced by CSE. (A) Western blotting demonstrated that AZM suppressed CSE-induced VEGF decreases. The data are presented as the mean ± standard error of the mean (n=3). ^*^P<0.05 versus controls; ^#^P<0.05 versus CSE. CSE, cigarette smoke extract; AZM, azithromycin; Con, control; VEGF, vascular endothelial growth factor; ZO-1, zona occludens-1; FITC, fluorescein isothiocyanate.

Our preliminary experiments revealed that AZM was important at 10 μg/ml in the alterations observed in VEGF, ZO-1 and occludin induced by CSE, which is also the physiological concentration, at 2 h prior to being incubated with CSE (data not shown) (30). Following incubation with SF-DMEM, A549 cells were pretreated with AZM (10 μg/ml) 2 h prior to being incubated with CSE. Following a 24 h period, VEGF was examined using western blotting. Downregulation of VEGF induced by CSE was attenuated by AZM at 10 μg/ml (Fig. 5A). In addition, western blotting and immunofluorescence analyses revealed that A549 cells exposed to CSE significantly decreased the expression of Occludin and ZO-1, while AZM inhibited these changes induced by CSE (Fig. 5A–C). These results indicated that AZM pretreatment for 2 h at a dose of 10 μg/ml was able to significantly reverse the changes in the expression of VEGF, Occludin and ZO-1 induced by CSE.

### NAC reverses the effect of CSE on occludin and ZO-1 expression and cell viability

**Figure 6:**
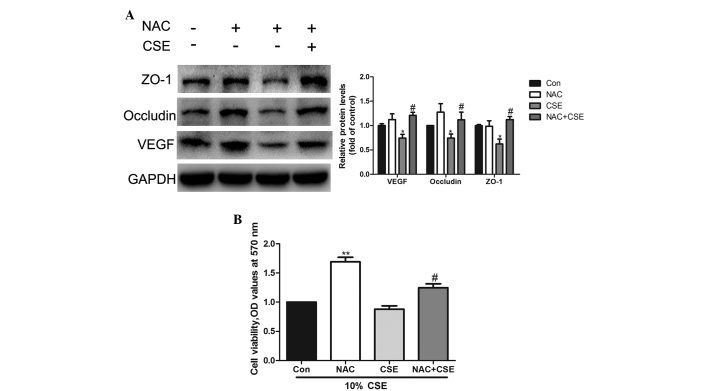
NAC reverses the effect of CSE on occludin and ZO-1 expression and cell viability. (A) Exposure of A549 cells to CSE significantly decreased the expression of occludin and ZO-1, while the oxidant scavenger NAC inhibited these changes induced by CSE. (B) Exposing A549 cells to NAC can increase cell viability. The data are expressed as the mean ± standard error of the mean (n=3). ^**^P<0.01 versus controls; ^#^P<0.05 versus CSE. CSE, cigarette smoke extract; VEGF, vascular endothelial growth factor; ZO-1, zona occludens-1; Con, control; OD, optical density; NAC, N-acetyl-L-cysteine.

The A549 cells were pretreated with 5 mM NAC (an antioxidant) for 2 h prior to CSE treatment. Western blot analysis demonstrated that NAC restores occludin and ZO-1 expression during CSE treatment (Fig. 6A). In addition, the data indicated that cell viability was increased by NAC (Fig. 6B).

## Discussion

CS is the main etiological factor contributing to respiratory disorders in the lung, including COPD and idiopathic pulmonary fibrosis (IPF) (7–9), which is characterized by the irreversible damage of lung epithelial cells. CS is a rich source of ROS with oxidative damage being the main pathogenic factor of CS (31). The chemical composition of CS is complex; therefore, it is not easy to predict which compounds or combinations of compounds may be involved in its effects. In the present study, an extract of CS, CSE, was used to imitate CS. Among the various cell types in the lung, AECs appear to be a major target for oxidant injury (2,3). It is well established that alveolar type II epithelial (AEC II) cells are stem cells of the alveolar epithelium (16,32). Following oxidant injury, the rapidity of initiation of AEC II proliferation is crucial for sufficient healing. A549 cells possess numerous features of AEC II cells (35) and a number of studies investigating CS-associated oxidant injury have used A549 cells as a model of AEC II (3,36–39). Therefore, in the present study, A549 cells were used as a model of AEC II, to examine the protective effects of AZM on CSE injury.

Excessive apoptosis of epithelial cells is an important factor in the pathogenesis of IPF and COPD. The results reported in the present study demonstrate that CSE affects A549 cell viability in a dose- and time-dependent manner, indicating that CSE has a deleterious effect on human AEC viability. A concentration of 10% CSE was selected for subsequent experiments as 10% CSE produced a maximal decrease in VEGF, ZO-1 and occludin proteins without apparent cell damage.

Lung epithelial barrier injury is one of the early pathological alterations induced by CS (5–7). Cell-cell junctions are important in maintaining cell and tissue polarity and integrity (40). Once the integrity of the epithelial barrier is damaged, opportunities for atmospheric components and pathogens to enter into the circulation and the interstitial and alveolar cavities increase, leading to the destruction of the alveolar walls and pulmonary edema (8,16). Epithelial integrity depends on the regulation of junctional complexes, including TJs. ZO-1 and occludin are markers of epithelial cells. When epithelial cells are damaged, typical features of the cell are disrupted and the expression of ZO-1 and occludin is decreased. A previous study demonstrated that the expression of ZO-1 decreased in smokers and patients with COPD (41). In animal models of acute lung and intestinal epithelial cell injury, TJ changes were associated with the downregulation of occludin and ZO-1 protein expression (42–44). In addition, ZO-1 and occludin are integral to TJs and CS may increase the permeability of AECs, which may be associated with the destruction of the TJ proteins (16). Numerous studies have revealed that ZO-1 and occludin expression alterations are associated with changes in the cytoskeleton (32–34). Additionally, the decrease of ZO-1 and occludin affects the connections between cells. Previous studies have demonstrated that AZM may increase transepithelial electrical resistance (45) and prevent disintegration of the TJ proteins in the airway epithelium exposed to *Pseudomonas aeruginosa* (46). AZM may inhibit epithelial-mesenchymal transition (EMT) in primary human small and large airway epithelial cells induced by transforming growth factor-β1 (47), causing cells to maintain their epithelial characteristics. However, whether AZM may reverse the alterations in ZO-1 and occludin proteins induced by CSE has not, to the best of our knowledge, been investigated previously. In the present study, it was observed that treatment with CSE significantly reduced ZO-1 and occludin expression in A549 cells in a dose- and time-dependent manner. It remains uncertain whether the reduction in ZO-1 and occludin and the protective effect of AZM is the result of changes in cell viability. As cell viability decreased, the expression of ZO-1 and occludin was reduced. However, it is unlikely that the decreased ZO-1 and occludin expression resulted from a direct cytotoxic effect of CSE as no significant reduction in viability of A549 cells was observed following incubation with 10% CSE for 24 h. The decreased ZO-1 and occludin expression was most likely secondary to CSE-induced alterations in cell structure. This conclusion is similar to that made when analyzing the effect of phenol on the barrier function of a human intestinal epithelial cell line (48) and glucose degradation products on human peritoneal mesothelial cells (49).

Smoking does not only affect the epithelial cell structure, but also the function of a series of epithelial cells. CS exposure is associated with reduced expression of VEGF and VEGF receptor (VEGFR)-2 in the lungs of patients with severe emphysema and in rodent lungs (21,50). In addition, the decrease in VEGF has been observed in the destruction of alveolar wall components, including microvasculature (20). Previous studies have suggested a beneficial role for VEGF in tissue repair and proliferation (51). VEGF may be an anti-apoptotic agent in the lung (52). The inhibition of VEGF results in increased markers of oxidative stress, alveolar enlargement and alveolar cell apoptosis in animals (53–55). VEGF is known to be a secreted protein (56). In our preliminary experiments, ELISA was used to measure the expression of VEGF. The results demonstrated that CSE attenuated the expression of VEGF in supernatant in a dose- and time-dependent manner. However, at 6 and 12 h, the level of VEGF was not able to be detected as VEGF did not reach the minimum concentration of the ELISA kits. The ELISA results were consistent with the western blotting results, suggesting that the content inside the cell is associated with exocrine function, therefore, in the preliminary experiments western blotting was used. The present study demonstrated that treatment with CSE significantly reduced VEGF expression in A549 cells in a dose- and time-dependent manner. It was also revealed that AZM pretreatment may reverse CSE-induced VEGF decreases for the first time, to the best of our knowledge. This indicated that AZM may be able to protect against epithelial injury induced by CSE. A possible explanation for this protective effect may be that AZM reduces the acute onset of COPD. AZM not only reversed the decrease in VEGF expression induced by CSE but also reduced the effects of CSE on epithelial cell integrity in the A549 cells, suggesting a protective response.

It has previously been reported that numerous chemical components in CS may induce ROS production (55). Oxidative stress is one of the classical signals of cell injury and may alter ZO-1 and occludin protein expression and localization (57,58). Taken together, the damaging effects of CSE on AECs may be initiated by oxidative stress. NAC is a well-known antioxidant (59) and pretreating A549 cells with 5 mM NAC for 2 h prior to exposure to CSE resulted in a significant increase in cell viability. In addition, western blotting indicated that the decreased expression of ZO-1 and occludin was reversed following NAC pretreatment. ROS are crucial factors that result in oxidative stress. Thus, augmentation of intracellular ROS may be one of the major factors contributing to CSE-induced injury in A549 cells. The present study demonstrated, for the first time to the best of our knowledge, that AZM decreases ROS generation induced by CSE. AZM, a macrolide antibiotic widely used in clinical practice, was reported to suppress neutrophil ROS release (27). In a cystic fibrosis airway epithelial cell line, AZM significantly reduced the activity of glutathione transferase (28). These findings indicate that AZM has antioxidant potential. However, whether AZM is able to inhibit the ROS generation induced by CSE in AECs has not been reported. CSE exposure caused oxidative stress, as revealed by the increased levels of ROS production. In the present study, CSE induced a significant increase in ROS levels. AZM pretreatment was used prior to CSE stimulation in A549 cells and it was observed that AZM may alleviate the changes in ROS production.

A number of controversial areas of this topic require further investigation. The establishment of functional TJs and the formation of impermeable monolayers in A549 cells has been questioned (60). However, a previous study demonstrated that the A549 cell line expresses ZO-1 with 16HBE14o-, a human bronchial epithelial cell line and Calu-3 cells in *in vitro* models and forms functional TJs (61). CS exposure has been associated with the increased permeability of A549 cells (62). In acute lung injury cell models exposed to thrombin, the permeability of A549 cells was hypothesized to be associated with ZO-1 and occludin (63). At the same time, as an epithelial marker, ZO-1 expression is decreased during EMT following injury (41) as the cells lose their epithelial cell character. These findings implicate the structural integrity of A549 cells associated with ZO-1 and occludin. In the present study, the cells formed confluent layers at the time of the experiments. It was demonstrated that the expression of ZO-1 and occludin is associated with the structural integrity of A549 cells. A structural change affects normal cell function. Thus, AZM has a similar protective effect on A549 cells. VEGF is a well-known permeabilizing factor and a downregulator of occludin and ZO-1 in vascular endothelial cells. The present study demonstrated that the change in VEGF is consistent with the expression of ZO-1 and occludin, which may involve more complex regulatory mechanisms requiring further investigation. Based on the present results, it is hypothesized that VEGF is expressed as several splice variants (64) and VEGFR also has different subtypes. The complex interactions may lead to the various downstream signaling pathways. Finally, although AZM protected A549 cells from the effects of CSE, whether this effect is true of human adult type I/II cells remains to be elucidated. Further investigation into the *in vivo* effects of AZM in animals is required to verify these findings.

In conclusion, the present study demonstrated that AZM is able to reverse smoke-induced aberrant expression of VEGF and structural proteins in A549 cells. Furthermore, AZM is able to inhibit the ROS production induced by CSE. Due to the widespread use of AZM in a clinical setting, its protective effect against oxidative stress caused by CS and as it is easily obtained, the present results may contribute towards new methods of treatment of smoking-associated disease, supporting that the present study has implications for clinical care.
